# Detection of low-frequency oscillations in neonatal piglets with speckle contrast diffuse correlation tomography

**DOI:** 10.1117/1.JBO.28.12.121204

**Published:** 2023-05-04

**Authors:** Mehrana Mohtasebi, Daniel Irwin, Dara Singh, Siavash Mazdeyasna, Xuhui Liu, Samaneh Rabienia Haratbar, Faraneh Fathi, Chong Huang, Kathryn E. Saatman, Lei Chen, Guoqiang Yu

**Affiliations:** a University of Kentucky, Department of Biomedical Engineering, Lexington, Kentucky, United States; b University of Kentucky, Spinal Cord and Brain Injury Research Center, Department of Physiology, Lexington, Kentucky, United States

**Keywords:** speckle contrast diffuse correlation tomography, cerebral blood flow, low-frequency oscillations, neuroimaging, neonatal piglet

## Abstract

**Significance:**

Low-frequency oscillations (LFOs) (<0.1  Hz) with respect to cerebral blood flow (CBF) have shown promise as an indicator of altered neurologic activity in the abnormal brain. Portable optical instruments have evolved to offer a noninvasive alternative for continuous CBF monitoring at the bedside compared with many large neuroimaging modalities. However, their utilization for acquiring LFOs of CBF has only been studied to a limited extent.

**Aim:**

We aim to optimize an innovative speckle contrast diffuse correlation tomography (scDCT) system for the detection of LFOs within CBF variations.

**Approach:**

The scDCT was optimized to achieve a higher sampling rate and a faster image reconstruction using a moving window 3D reconstruction algorithm with parallel computation. Power spectral density (PSD) analysis was performed to investigate altered LFOs during transient global cerebral ischemia in neonatal piglets.

**Results:**

Transient global cerebral ischemia resulted in reductions in both CBF and PSD compared with their baseline values.

**Conclusions:**

Spontaneous LFOs, combined with CBF, provide a more comprehensive assay with the potential to clarify pathological mechanisms involved in brain injury. These results support scDCT’s inclusion and application in the growing area of LFO analysis and demonstrate its inherent advantage for neurological studies in preclinical and clinical settings, such as neonatal intensive care units.

## 1 Introduction

The brain is one of the most complex and metabolically demanding organs in humans. In spite of its relatively small size, the brain consumes ∼20% of the oxygen in the body.^1^ The function of the brain depends on cerebral blood flow (CBF) to deliver oxygen and glucose and to clear metabolic wastes. Blood pressure, neurogenic activity, and cardiac output all contribute to regulating CBF.^2^ Rhythmic oscillations present in CBF are driven by the cardiac cycle, respiratory motion, myogenic, neurogenic, and metabolic mechanisms.^3^ Analysis of these oscillations reveals individual physiological contributions that arise at unique frequencies. In humans, ∼1 and ∼0.3  Hz oscillations are associated with heartbeat and respiration, respectively, and those below 0.1 Hz are generally termed low-frequency oscillations (LFOs). LFOs also refer to Mayer waves.^3^^–^^5^ Heartbeat and respiratory rates scale with the size of the species.^6^ On the other hand, LFOs have not been shown to be dependent on size as studies have demonstrated that, in piglets,^7^ monkeys,^8^ cats,^9^^,^^10^ rabbits,^11^ and rodents,^6^^,^^12^^,^^13^ most LFOs exist below 0.1 Hz, which is the same frequencies as found in humans.^3^^–^^5^^,^^13^ This feature makes LFOs a potentially attractive target for producing translatable neurological findings.

Central nervous system disease or injury can result in an abnormal CBF status and disruption to cerebral autoregulation. The cerebral vasculature network is affected by pathological disturbances, which impair dynamic oxygen and energy supply of neurons,^14^ thereby interfering with distributed patterns of synchronized neural activity throughout the brain.^15^ An increase in CBF (hyperperfusion of the brain) may result in increased intracranial pressure, headaches, and intraventricular hemorrhage (IVH). Insufficient CBF (hypoperfusion of the brain) may lead to the depression of cerebral function as well as ischemic stresses on cerebral tissues. Such CBF abnormalities also impact rhythmic oscillations, including LFOs. Specifically, LFOs remain relatively stable in healthy brains, whereas changes in local LFOs are associated with compromised brain tissue.^16^ Therefore, it is important to continuously monitor LFOs to gain a better understanding of pathological mechanisms underlying these diseases and to develop medical interventions.^17^ Moreover, the quantification of temporal correlations in LFOs across different regions of the brain [i.e., mapping functional connectivity (FC)] enables detecting regional neurovascular disorders.^18^ There is then an unmet need for continuous neuroimaging tools that offer sufficiently high temporal and spatial resolutions to accurately detect LFOs and generate FC maps.

A few noninvasive or minimally invasive neuroimaging tools capable of measuring cerebral hemodynamics are available for LFO applications, such as functional magnetic resonance imaging (fMRI) and optical methods. Blood-oxygen-level-dependent fMRI studies have revealed abnormal LFO amplitudes in patients with schizophrenia,^19^ mild cognitive impairment,^20^ Alzheimer’s disease,^21^ major depressive disorder,^22^ sleep-deprived brain,^23^ traumatic brain injury,^24^ and stroke,^25^ and it has been utilized for cerebral FC analysis in the study of cognitive neurosciences and clinical psychiatry/psychology.^26^^,^^27^ Despite these successes with fMRI, technical and logistical challenges remain; these include low temporal resolution, motion artifact, electromagnetic compatibility, high cost, and lack of portability.^28^

Optical imaging techniques offer noninvasive, fast, inexpensive, and portable means for cerebral hemodynamic and LFO assessments. Wide-field optical illumination with laser speckle contrast imaging^29^^–^^31^ and optical intrinsic signal imaging (OISI)^32^^,^^33^ map two-dimensional (2D) distributions of CBF and cerebral oxygenation, respectively. Their penetration depth is limited to <1  mm, restricting their use to the superficial cortex in rodents and precluding their use for noninvasive cerebral imaging in larger animals such as piglets and in humans. Near-infrared (NIR) techniques, such as near-infrared spectroscopy (NIRS) and diffuse correlation spectroscopy (DCS), allow for continuous, noninvasive measurements of cerebral oxygenation and CBF, respectively, in deep brain tissues (up to centimeters).^34^^,^^35^ For example, DCS was used to assess LFO amplitudes as a biomarker of neurologic injury during the acute period after cardiac arrest in a piglet model of pediatric cardiac arrest.^7^ Correlations between the amplitudes of LFOs of CBF and the invasive biomarkers of neurologic injury obtained from microdialysis were found during the first 4 h following cardiac arrest. Results from this study demonstrate that LFO is a promising biomarker for evaluating neurometabolic dysfunction in the postresuscitation period. Interests in the study of LFOs in human subjects have also been increasing. For example, a hybrid NIRS/DCS instrument has been used to simultaneously detect LFOs of CBF, oxy-hemoglobin concentration ($\left[{\mathrm{HbO}}_{2}\right]$), and deoxy-hemoglobin concentration ([Hb]) in cognitively healthy older subjects with high or low risk for developing cerebrovascular disease.^35^^,^^36^ The LFO amplitudes in the high-risk group were lower than those in the low-risk group.^36^ Studies using functional NIRS^37^^,^^38^ have shown impairments in brain FC maps^39^^,^^40^ and LFO amplitudes^41^ in patients with cerebrovascular diseases compared with healthy controls. Using high-density diffuse optical tomography (HD-DOT) with numerous sources and detectors,^16^^,^^42^^,^^43^ abnormal amplitudes of LFOs and FC maps of cerebral oxygenation in occipital cortices were obtained from premature infants.^16^

Although these diffuse optical systems show promise for detecting brain LFOs, there remain several limitations. NIRS/DCS systems suffer from limited numbers of discrete light sources and detectors, thereby taking sparse source–detector (S–D) pair measurements over a region of interest (ROI). This limits spatial resolution^44^ and head coverage, thus inhibiting the localization of activations from regionally distributed brain functions.^45^ Cerebral signals collected using NIRS/DCS methods are also inherently influenced by partial volume effects from overlayer tissues (scalp and skull),^35^^,^^36^ causing difficulty in discriminating the brain signal of interest. HD-DOT systems in particular have received much attention with regards to improved spatial resolution and brain signal differentiation.^16^^,^^43^^,^^46^ Nonetheless, extending the field of view (FOV) with fiber-based HD-DOT contact measurement systems to gain a more complete view of the brain is hampered by complications with optical fiber coupling to heads and cap design, especially for the heads of small and fragile newborn infants.^16^^,^^43^^,^^46^

Recently developed speckle contrast diffuse correlation tomography (scDCT) (US Patent No. 9/861,319, 2018^47^) technology provides a noninvasive, fully noncontact, low-cost, and portable tool for high-density 3D imaging of blood flow distributions in deep tissue volumes (up to ∼10  mm).^48^^–^^50^ Importantly, this device remedies many previous limitations while offering direct CBF imaging for potential LFO assessments. In contrast to the NIRS, DCS, and HD-DOT systems using discrete detectors, scDCT uses a complementary metal-oxide semiconductor (CMOS) camera with thousands of pixels to serve as parallel 2D detectors, which dramatically increases the sampling density and enables a wide FOV. The light source is directed to an electronic-controlled galvo-mirror, which can rapidly scan the FOV with flexible patterns of desired source density. These benefits are combined with a unique finite-element-method (FEM)-based reconstruction technique capable of incorporating complex surface geometries and known tissue properties.^51^^–^^53^ Moreover, noncontact scDCT manages large numbers of sources and detectors and alleviates the need for complicated head helmet design, thus resulting in straightforward applications on subjects with different scales/sizes including small animals and preterm infants. In fact, case studies have already successfully demonstrated the scDCT usage for continuous and longitudinal imaging of CBF distributions in tissue phantoms; *in vivo* cerebral tissues of rodents undergoing ${\mathrm{CO}}_{2}$ inhalation and unilateral/bilateral ligations of common cerebral artery (CCA); neonatal piglets with CCA ligations and IVH; and sleeping preterm infants without scalp preparation through a transparent incubator wall.^48^^,^^54^^–^^57^ Although these applications illustrate scDCT capability in identifying localized CBF responses across many subject sizes, the cited studies did not incorporate the detection of LFOs.

The goal of this study is to optimize the scDCT system for the detection of LFOs within CBF variations. Neonatal piglets were selected as model subjects due to their mid-range head size, high anatomical similarity, and translational potential to human neonates, as well as our recent success with original scDCT techniques for comparisons.^48^ The numbers of scanning sources and detectors in the scDCT were optimized to balance temporal and spatial resolutions of CBF imaging to examine regional brain responses and derive LFOs after signal filtering. A moving window 3D reconstruction method was adapted to achieve a fast sampling rate of 5 Hz, which is much higher than that with conventional reconstruction method (e.g., 0.2 Hz).^48^^,^^58^ This sampling rate (5 Hz) is faster than typical cardiac and respiratory rates in neonatal piglets (<2.5  Hz), thus allowing for the removal of their contributions to CBF signals using simple lowpass filters. Moreover, the reconstruction of a single 3D image in previous studies took minutes to hours depending on the size of mesh nodes.^48^^,^^53^^,^^59^^,^^60^ In response, a new 3D reconstruction algorithm using parallelization functionalities in MATLAB (MathWorks) was developed to achieve a sizable increase in reconstruction speed while maintaining reconstruction quality. As a result, the computation time for 3D reconstruction in this study was reduced from 15 min to 50 s. With these notable improvements, LFOs in piglet brains were successfully isolated and quantified by the optimized scDCT technique. This pilot preclinical study served as a vital and straightforward step toward using scDCT to map cerebral FC maps in human newborns.

## 2 Materials and Methods

### 2.1 Animal Preparations and Experimental Protocols

All experimental protocols in animals were approved by the Institutional Animal Care and Use Committee at the University of Kentucky. Two male neonatal piglets (age: 32-days old) were purchased through the Division of Laboratory Animal Resources from the College of Agriculture Swine Center at the University of Kentucky. Their genetic background included 62.5% Landrace, 25% Large White, and 12.5% Yorkshire. The two piglets were housed together in one dedicated room in a pigpen, with the support of a heat lamp and towel-covered floor. They were fed ad libitum with milk replacer (Birthright, Ralco) for the first postnatal week and gradually transitioned to pellet food (Ignite Pre-Starter, Ralco) at 2 to 3 weeks.

**Fig. 1:**
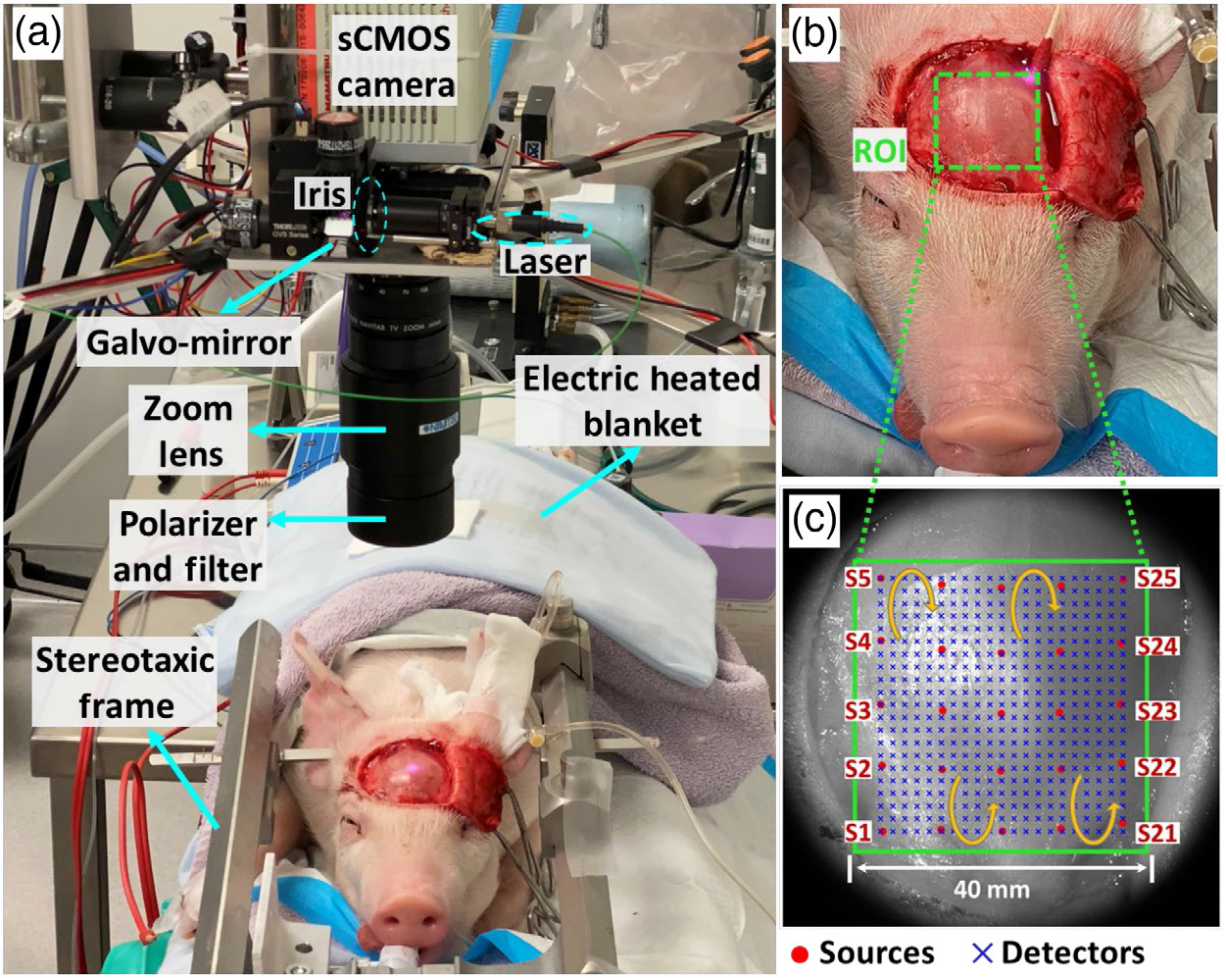
Experimental setup for continuous CBF monitoring of neonatal piglets during transient global ischemia. (a) The anesthetized piglets were secured on a stereotaxic frame. A respirator-oximeter probe was clipped on the ear to acquire BPM, RPM, ${\mathrm{EtCO}}_{2}$, and ${\mathrm{SpO}}_{2}$. The noncontact scDCT was set up overhead and used for 3D imaging of rCBF distributions. (b) For scDCT, an ROI of $40\times 40\;{\mathrm{mm}}^{2}$ was projected on the intact skull of piglet head without the need for an invasive transcranial window. (c) The ROI was covered by evenly distributed 5×5 sources (scanned) and 21×21 detectors (grouped pixels).

To study LFO variations, transient global cerebral ischemia was induced in the two neonatal piglets (Fig. 1). The piglets were fasted for at least 4 h before surgery to avoid reflux of stomach contents. Piglets were intubated with a pediatric endotracheal tube (internal diameter: 3 mm) and maintained under anesthesia with 1.5% to 2% isoflurane. Following previous studies in neonatal piglets,^48^ the anesthetized animal was placed prone and its head secured on a customized stereotaxic frame. The animal’s body was wrapped with an electric heated blanket under the surveillance of a rectal thermometer to avoid hypothermia. A multichannel respirator-oximeter sensor (8400, Smiths Medical) was used to record heartbeat per minute (BPM), respiration per minute (RPM), end-tidal carbon dioxide (${\mathrm{EtCO}}_{2}$), and peripheral artery blood oxygen saturation (${\mathrm{SpO}}_{2}$).

Both piglets underwent transient unilateral and bilateral CCA ligations to create sequential decreases in CBF within the left and right cerebral hemispheres. After hairs on the head and ventral cervical skin were shaved with a clipper and cleaned with hair cream, the skin was disinfected with betadine and wiped with 70% ethanol. The animal was laid supine, and a 5-cm midline incision was made in the cervical area. The skin was retracted with four customized surgical hooks for better exposure of the surgical area. The underlying tissue was separated with blunt dissection until both CCAs were exposed. A 6-0 braided nylon suture was placed around each CCA, and a loose knot was made on each suture without disrupting the blood flow to the brain. The piglet was then laid prone. A $50\times 50\;{\mathrm{mm}}^{2}$ C-shape incision was made on the scalp to expose the skull. After a baseline measurement of scDCT for 5 min, the right knot was tightened to occlude the right CCA for ∼5  min, followed by tightening the left knot for ∼2  min to induce a transient global cerebral ischemia (i.e., both right and left CCA occlusions). The left and right knots were then released sequentially, allowing for the restoration of the blood flow to the brain (i.e., recovery phase). After the restoration of CBF from transient global cerebral ischemia, the piglet was euthanized with 100% ${\mathrm{CO}}_{2}$ for ∼4  min.

### 2.2 scDCT System for Data Acquisition

Details about scDCT techniques for blood flow measurements are available in our previous publications.^48^^,^^52^^,^^54^^,^^55^^,^^57^^,^^60^^–^^62^ Briefly, a high-speed scanning galvanometer mirror positioning system (maximum scan angle: ±12.5  deg, switching time <1  ms, GVS002, Thorlabs) remotely and sequentially delivered coherent focused-point NIR light (780 nm, CrystaLaser) to multiple source positions on the tissue surface for boundary data acquisition [Fig. 1(a)]. An adjustable iris diaphragm (SM05D5, Thorlab) was placed into the source path to optimize the intensity and spot size of the incident light. A scientific CMOS (sCMOS) camera (pixels: 2048×2048; frame rate: 30/s; quantum-efficiency: 50% at 800 nm, ORCA-Flash4.0, Hamamatsu Photonics) was used as a high-density 2D detector array to collect re-emitted light from the tissue for measures of spatial diffuse speckle contrasts in a selected ROI. The movement of red blood cells in the measured tissue volume produced spatial fluctuations of laser speckles captured by the sCMOS camera with a typical exposure time of 2 ms. A zoom lens (Zoom 7000, Navitar) was connected to the camera for adjusting the size of the ROI [Fig. 1(b)]. A long-pass filter (>750  nm, #84-761, EdmundOptics) was installed in front of the zoom lens to reduce the impact of ambient light. A pair of polarizers were added across the source and detection paths to reduce specular reflection directly from the scanning light sources on the tissue surface.

The noncontact scDCT system was adjusted above the piglet to cover an ROI of $40\times 40\;{\mathrm{mm}}^{2}$ on the head after skull exposure and before CCA occlusion commencement. The continuous data acquisition procedure consisted of sequentially scanning a set of 5×5 source locations on the ROI in a cycling fashion [Fig. 1(c)]. The total sampling time for scanning over 25 source positions was 5 s (0.2 s per source position). For each source position, the sCMOS camera, as a 2D detector array, output a raw intensity image.

The power of incident point light that reached tissue surface from the scDCT was <0.5  mW, according to the measurement by a power meter. This low level of power is considered safe according to the American National Standards Institute standard under the Accessible Emission Limit Class 3R classification.^55^ Moreover, the eyes of the anesthetized piglet were always closed during scDCT measurements. Thus the point light scanned over the selected ROI on the head and never shined on the eyes. Nonetheless, further eye protection will be made in the future by placing a protective eyewear on their eyes.

### 2.3 Boundary Data Processing for CBF

**Fig. 2:**
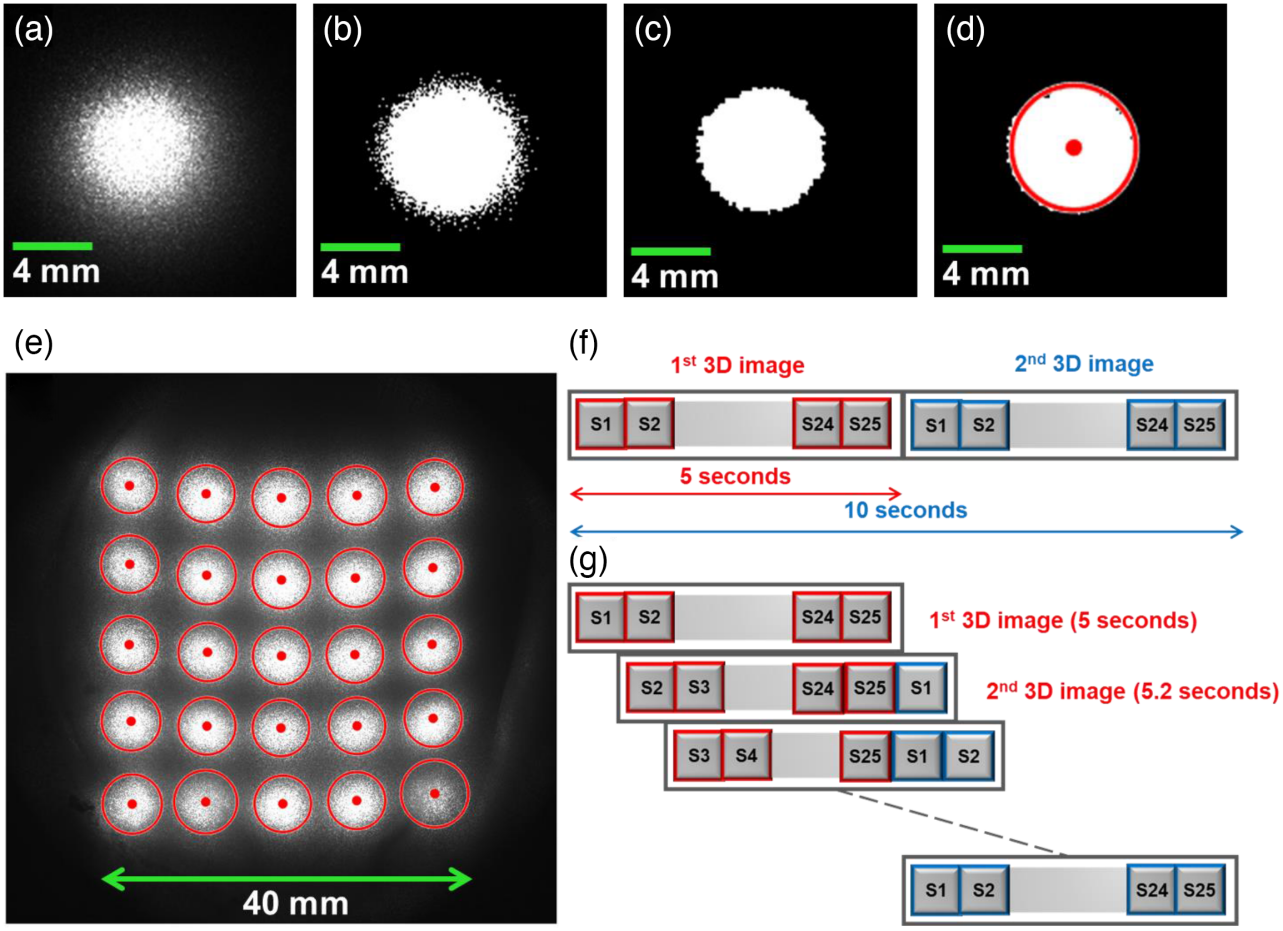
scDCT image processing for determining source locations and reconstruction steps for generating 3D CBF images. (a) A raw image captured by the camera. (b) The raw image from (a) converted to a binary image, derived by replacing all values above a globally determined threshold with “1” and setting all other values to “0.” (c) The object from (b) with the maximum number of connected pixels where morphological operators were applied to remove the noise from the image. (d) The centroid and radius of the point source was determined by estimating the center of mass for the remaining object in (c). This process was performed on all raw images to determine each source location. (e) Twenty-five raw images with their centroid location were added for presentation purposes. (f) Conventional reconstruction paradigm: each 3D CBF reconstruction was performed after fully updated boundary data were available (every 5 s). (g) Moving window reconstruction method: the first 3D image reconstruction took 5 s. However, a new 3D CBF image was then produced as soon as the raw image from one new source position was available, which occurred approximately every 0.2 s.

For the 3D reconstruction of CBF, source and detector positions on the mesh surface must be supplied. To precisely find the actual source location, the intensity image [Fig. 2(a)] was first converted to a binary image [Fig. 2(b)]. This was done by replacing all intensity values above a globally determined threshold with “1” and setting all other values to “0.” The threshold value was determined by Otsu’s method to minimize the intraclass variance of the thresholded black and white pixels.^63^ After thresholding, those thresholded pixels with a value of “1” that connected to 8 pixels in local neighborhoods (so called 8-connected) were found and grouped together. In this automatic process, the “bwlabel” built-in function in MATLAB was employed to label the connected components in the binary image. By setting the “conn” parameter in the bwlabel function to 8, eight neighboring pixels were determined. Then the “regionprops” built-in function with the “area” property was applied to compute the connected area, labeled as pixel numbers. The area with the maximum number of pixels was identified using the max function in MATLAB and other areas were then removed from the 2D binary image. Morphological operators, such as opening, closing, and filling, were then applied to the binary image for removing thin protrusions and isolated areas to reduce noises [Fig. 2(c)]. Finally, the centroid and radius of the point source were determined by estimating the center of the remaining largest area using the regionprops function with the “centroid” property [Fig. 2(d)]. This automatic process was repeated for all source locations [Fig. 2(e)].

After finding these source locations on the X−Y plane, 441 detectors (21×21) were then evenly distributed between the 25 sources in the selected ROI of $40\times 40\;{\mathrm{mm}}^{2}$ to cover both hemispheres [Fig. 1(c)]. With this S–D configuration, the distance between two adjacent detectors was 2 mm. This configuration allowed for arranging 441 detectors on the ROI without any overlap and with balance between the spatial resolution and computation time. Moreover, S–D pairs with signal-to-noise ratios (SNRs) below 3 (linear scale) or above 90% of saturated intensity were excluded from the data analysis. The SNR was calculated by the ratio of $I/I_{\text{noise}}$, where I and $I_{\text{noise}}$ denote the detected light intensity and dark noise of the camera, respectively.

Another processing step was to extract boundary blood flow indices (BFIs) from measured speckle contrasts in the raw intensity images. The speckle contrast relies on the statistical property of spatial signal intensity distribution and was quantified by calculating the intensity ratio of standard deviation (σ) to mean (⟨I⟩), i.e., K=σ/⟨I⟩ in a pixel window of 7×7  pixels on the sCMOS camera. We then averaged K values over 3×3 adjacent pixel windows with an area of $0.2\times 0.2\;{\mathrm{mm}}^{2}$ (as a single detector out of total 441 detectors) to improve SNRs. These speckle contrast calculations were carried out at the effective S–D distances of 6 to 16 mm, which corresponded to penetration depths up to ∼8  mm (i.e., one half of the maximal S–D separation). Boundary BFI data were then obtained through a nonlinear relation with K. The derivation of this nonlinear relation can be found in our previous publication.^52^ The boundary BFI at one S–D pair was extracted by iteratively minimizing the difference between the measured and theoretical speckle contrast, with a total computation time of ∼100  ms.

### 2.4 3D Image Reconstruction of CBF

The S–D pair coordinates, their boundary BFIs, and a solid volume tetrahedral mesh with a 30 mm height, $60\times 60\;{\mathrm{mm}}^{2}$ bottom area, and 20k nodes were input into our FEM-based reconstruction program, i.e., modified NIR fluorescence and spectral tomography, to generate a complete 3D image of CBF distributions in the measured tissue volume.^51^^–^^53^ The relative change in CBF (rCBF) was calculated by normalizing the reconstructed BFI to its corresponding baseline before physiological manipulations.

In the conventional reconstruction method, blood flow distribution in the measured tissue volume is reconstructed once a full source scanning cycle is completed. In other words, the intensity images for all source locations (i.e., 5×5 sources) should be available to generate a complete tomographic flow image [Fig. 2(f)]. Then the process is repeated for subsequent scanning cycles (i.e., another 5×5 sources). As a result, the total scanning time in previous studies using 5×5 sources was 5 s (0.2 Hz). Decreasing the number of source positions would improve the temporal resolution. However, this would undesirably reduce the sampling density and SNRs. Alternatively, we implemented a new reconstruction method based on a moving window, in which a tomographic reconstruction was obtained each time one new source position became available [Fig. 2(g)].^58^ The moving window method updated data at each new source position every 0.2 s. As a result, the sampling rate of the moving window method (5 Hz) was 25 times higher than the conventional reconstruction method (0.2 Hz). Using this higher sampling rate (5 Hz), carrier frequencies from cardiac and respiratory variations are unlikely to be aliased into LFO bands and can be removed with simple low-pass filters. Typical heartbeat and respiratory rates for piglets depend on their age and the anesthetic used. In our study, the heartbeat and respiratory rates for piglets were around 2.5 and 0.5 Hz, respectively.

The reconstruction procedure itself is often an expensive task with a high computational overhead. The higher sampling rate achieved in this study carried a greater burden of reconstructing for all time points. With original reconstruction procedures, processing such a magnified dataset size could prohibit the practical use and longitudinal applications. To improve the speed of reconstructing multiple images, we parallelized the for-loop iterations using MATLAB’s parfor control flow statement available in the Parallel Computing Toolbox™. This was possible because the 3D reconstructions of multiple rCBF images were independent, thus allowing for the execution of iterative calculations simultaneously. Our approach utilized powerful computers at the University of Kentucky High-Performance Computing (HPC) Center to achieve fast parallel computations of Jacobian matrices across multiple S–D pairs and iterative solvers.^51^^–^^53^ The computer configuration was Intel^®^ Xeon^®^ Gold 6252 high-performance server microprocessor, 16 CPU cores at 3.70 GHz (max turbo frequency). For a single 3D image reconstruction of CBF distribution in the slab mesh with total mesh nodes of 20k as used in this study, the sublimated speed and memory efficiency led to 18 times reduction of computation time (from 15 min to 50 s).

### 2.5 Power Spectral Density Analysis for LFO Intensity

The PSD analysis for LFO intensity quantification has been established in diffuse optical technologies including NIRS^16^^,^^64^ and DCS.^7^^,^^35^^,^^36^ Following these methods, LFO intensities of CBF under different physiological conditions (i.e., resting baseline, right CCA occlusion, transient global ischemia, releasing left CCA occlusion, and sequentially releasing right CCA occlusion for recovery) were extracted from PSDs calculated by Welch’s method in a nonparametric approach.^35^ Specifically, the PSD was calculated as follows: $\mathrm{PSD}=1/(F_{s}\times N_{\mathrm{seg}})\sum |{\mathrm{FFT}}_{X_{\mathrm{seg}}}(f){|}^{2}$, where $F_{s}$ is the sampling frequency, $N_{\mathrm{seg}}$ is the number of segments, and ${\mathrm{FFT}}_{X_{\mathrm{seg}}}$ is the fast Fourier transform of the segmented signals.^65^ Briefly, the time-course rCBF data under each physiological condition at the sampling rate of ∼5  Hz (frame per second) were first detrended using a built-in “detrend” function in MATLAB to obtain the best straight-line fit linear trend. The detrended rCBF data were then transferred into a Butterworth filter with a passband at the LFO range from 0.0 to 0.1 Hz. Finally, the built-in “pwelch” function in MATLAB was used to generate the PSD over the LFO bandwidth.

To compare PSD levels across different phases of transient global cerebral ischemia, PSD at each phase was normalized to the AUC of the baseline PSD. The trapezoidal integration method in MATLAB built-in “trapz” function was used to calculate the AUC of PSD. The half-width-at-half-max (HWHM) value was then determined from the normalized PSD by quantifying the half width of the frequency band, in which the PSD was above half of its maximum value.

## 3 Results

### 3.1 rCBF and Vital Variations During Transient Global Ischemia

**Fig. 3:**
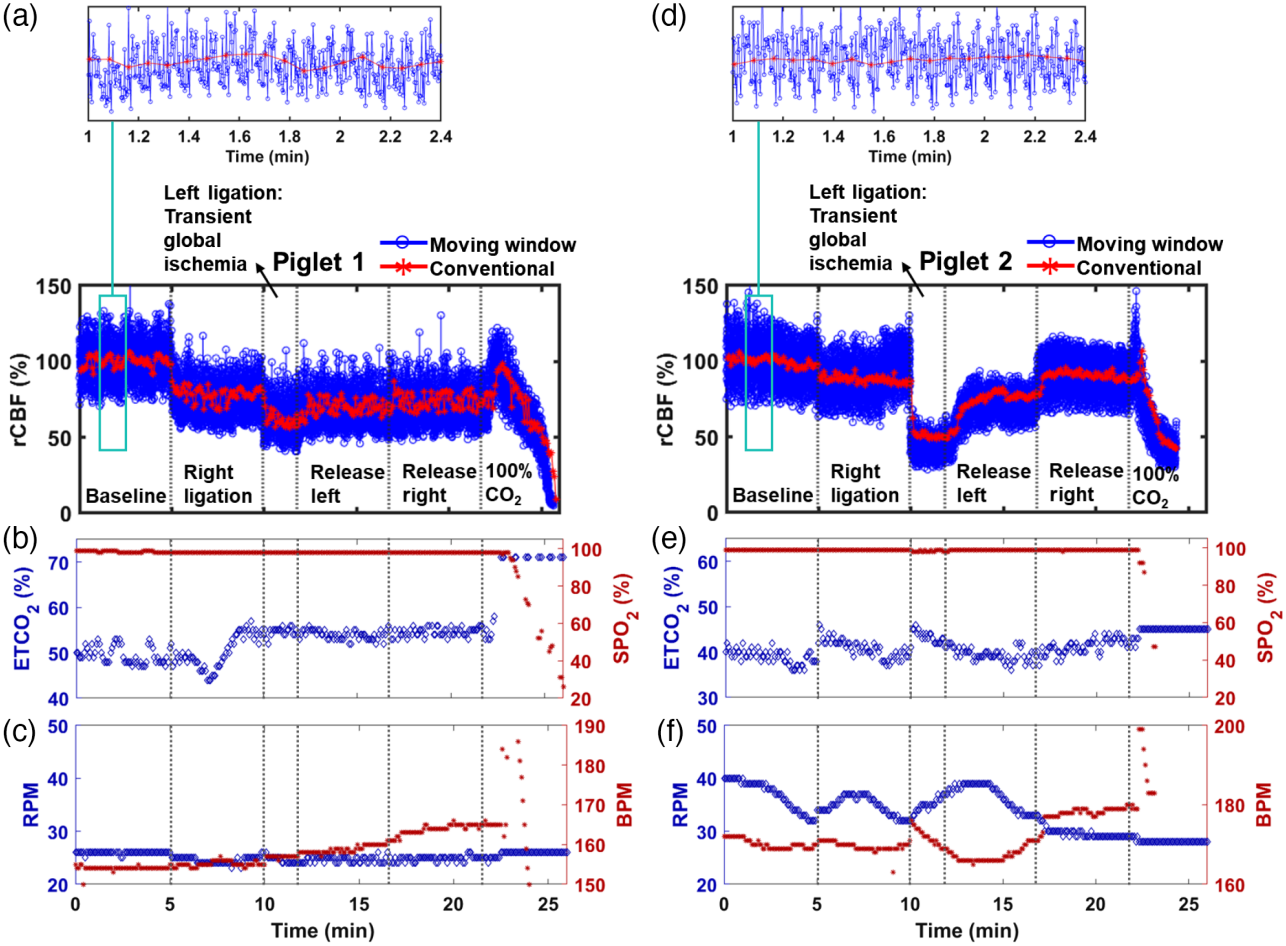
Measurement results from piglet #1 and piglet #2 during transient global ischemia. (a), (d) Time-course changes in rCBF measured by scDCT at 5-mm depth during sequential unilateral and bilateral CCA ligations. The experimental protocol included the baseline, right CCA ligation, bilateral ligation (transient global ischemia), releasing of left ligation, releasing of both left and right ligations (recovery), and euthanasia (100% ${\mathrm{CO}}_{2}$). Red curves indicate the time points at which a 3D CBF reconstruction was available with the conventional reconstruction method, and blue curves indicate the time points with the moving window method. Two zoom-in magnified areas are shown to highlight the increased temporal resolution of the rCBF by the moving window method (blue). Time-course changes in (b) ${\mathrm{EtCO}}_{2}$, (e) ${\mathrm{SpO}}_{2}$, (c) RPM, and (f) BPM.

Figure 3 shows physiological changes measured by the scDCT and multichannel respirator-oximeter during transient global ischemia in two neonatal piglet brains following the CCA occlusion protocol. Figure 3(a) shows rCBF data averaged over the ROI at the depth of 5-mm beneath the skull of piglet #1, quantified by the moving window (blue curve) and conventional (red curve) reconstruction methods, respectively. Based on neonatal piglet head anatomy, the depth of 5 mm reaches the brain cortex.^48^ During right CCA occlusion, rCBF declined, reaching 75%±11% and 79%±4% (mean ± standard deviation in timeseries of rCBF values) of the baseline from the moving window and conventional reconstruction methods, respectively. Bilateral ligation further decreased rCBF to 63%±11% (moving window) and 63%±6% (conventional reconstruction), respectively. After sequentially releasing left and right ligations, rCBF values were 68%±11% (moving window)/70%±5% (conventional reconstruction) and 70%±12% (moving window)/74%±5% (conventional reconstruction), respectively. At the end of the experiment, euthanasia was induced by $100\%{\mathrm{CO}}_{2}$ inhalation until the heartbeat stopped. rCBF had an immediate but brief reactive hyperemic response and then dropped to a minimum of 7% (moving window)/9% (conventional reconstruction) by the conclusion. As expected, both the moving window and conventional reconstruction methods portrayed similar trends over all protocol phases.

Figure 3(d) shows time-course changes in rCBF detected by scDCT during transient global ischemia in piglet #2. Compared with piglet #1, more pronounced decreases occurred during bilateral ligation compared with unilateral ligation. Specifically, rCBF declined to 90%±12% (moving window)/88%±2% (conventional reconstruction) of the baseline during right CCA ligation and to 45%±7% (moving window)/53%±8% (conventional reconstruction) during bilateral ligation. Then rCBF increased to 68%±11% (moving window)/72%±9% (conventional reconstruction) during release of left ligation and 91%±11% (moving window)/90%±3% (conventional reconstruction) during release of right ligation, respectively. Transient reactive hyperemic responses were detected during the induction of euthanasia by $100\%{\mathrm{CO}}_{2}$. rCBF dropped to 30% (moving window)/42% (conventional reconstruction) during the euthanasia, which were less dramatic than those observed in piglet #1.

To summarize rCBF results from the two piglets, the model of sequential CCA ligations enabled the prompt manipulation of rCBF. Right CCA ligation induced an instant reduction in rCBF and sequential bilateral occlusions of CCA resulted in a transient global cerebral ischemia. Sequential release of left and right CCAs allowed for the restoration of rCBF. These changes in rCBF are expected and consistent with previous findings in rodents and neonatal piglets during transient global cerebral ischemia.^48^^,^^54^^,^^57^ Although both methods showed similar trends, the moving window method with a higher sampling rate demonstrated larger variations in rCBF, which are likely associated with other physiological changes, such as heartbeat and respiration rates. Importantly, the high sampling rate of the moving window method enabled the detection of LFOs without any interference from heartbeat and respiration.

Fluctuations of ${\mathrm{EtCO}}_{2}$, ${\mathrm{SpO}}_{2}$, RPM, and BPM during transient global ischemia are shown in Figs. 3(b) and 3(c) for piglet #1 and Figs. 3(e) and 3(f) for piglet #2. The increased ${\mathrm{CO}}_{2}$ concentration (${\mathrm{EtCO}}_{2}$, blue dots in Figs. 3(b) and 3(e)] along with the reduced $O_{2}$ concentration during euthanasia inhibited respiratory and cardiac functions, thus leading to large drops in rCBF [Figs. 3(a) and 3(d)], ${\mathrm{SpO}}_{2}$ [red dots in Figs. 3(b) and 3(e)], and BPM [red dots in Figs. 3(c) and 3(f)]. Piglet #2 was found to have a marginal change in ${\mathrm{EtCO}}_{2}$, which is consistent with the higher observed rCBF minimum upon euthanasia compared with piglet #1. Unfortunately, data were not collected from the respirator-oximeter device during euthanasia of piglet # 2 due to a technical problem with the device, but a BPM decline was confirmed by measuring the heartbeat with a stethoscope. The RPM [blue dots in Figs. 3(c) and 3(f)] response in piglet #1 was negligible, whereas that in piglet #2 displayed a slow decrease throughout. These physiological variations resulting from the hypoxic challenge agreed with pathophysiological processes.

### 3.2 LFOs During Transient Global Ischemia

**Fig. 4:**
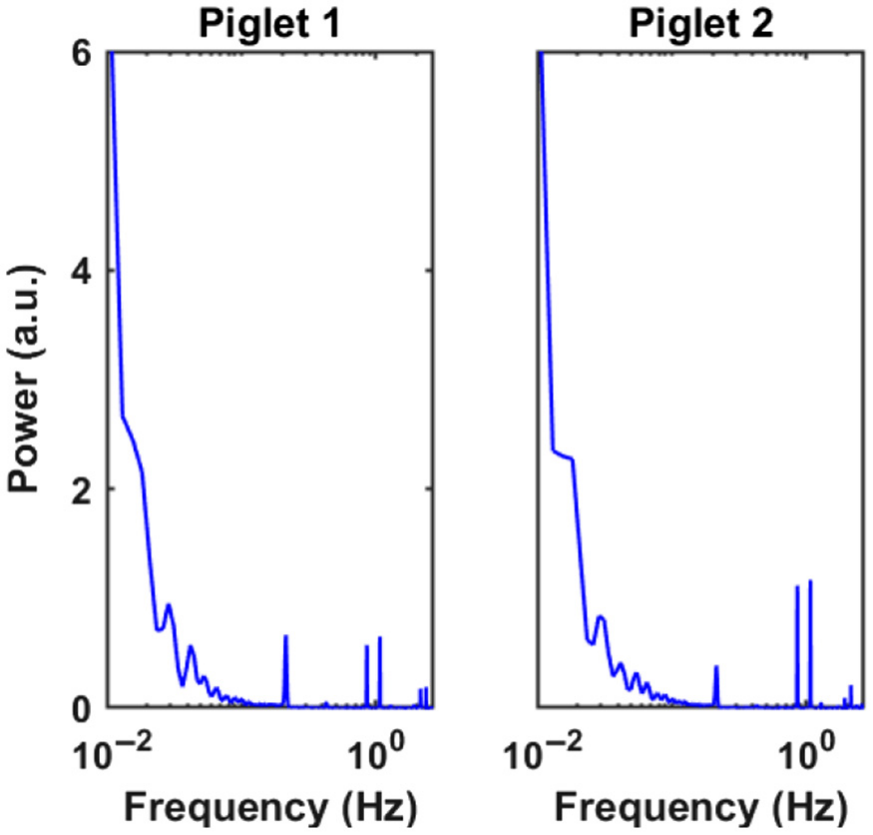
Power spectra of rCBF signals during baseline measurements. Power spectra were determined from reconstructed rCBF at 5 mm beneath the skull as acquired using scDCT with the moving window reconstruction method. Baseline measurements preceded physiological manipulations (i.e., CCA ligations). LFOs (<0.1  Hz), heartbeat (2.5 Hz), and respiratory rate (0.5 Hz) were captured.

LFOs were isolated from moving window rCBF curves [Figs. 3(a) and 3(d) blue curves] for each session of physiological conditions. The LFO range (<0.1  Hz) was identified through the power spectra of rCBF as shown in Fig. 4. rCBF signals were taken from the baseline measurement interval before physiological manipulations. With the new moving window method, scDCT achieved a sampling rate of 5 Hz, which was sufficient for capturing the heartbeat (2.5 Hz) and respiration rate (0.5 Hz). Apparently, high-frequency signals arising from respiration and cardiac cycles were aliased into the desired LFOs. Using a low-pass filter, rCBF signals were reanalyzed for LFO isolation while filtering the artifactual signals unrelated to neural activity.

**Fig. 5:**
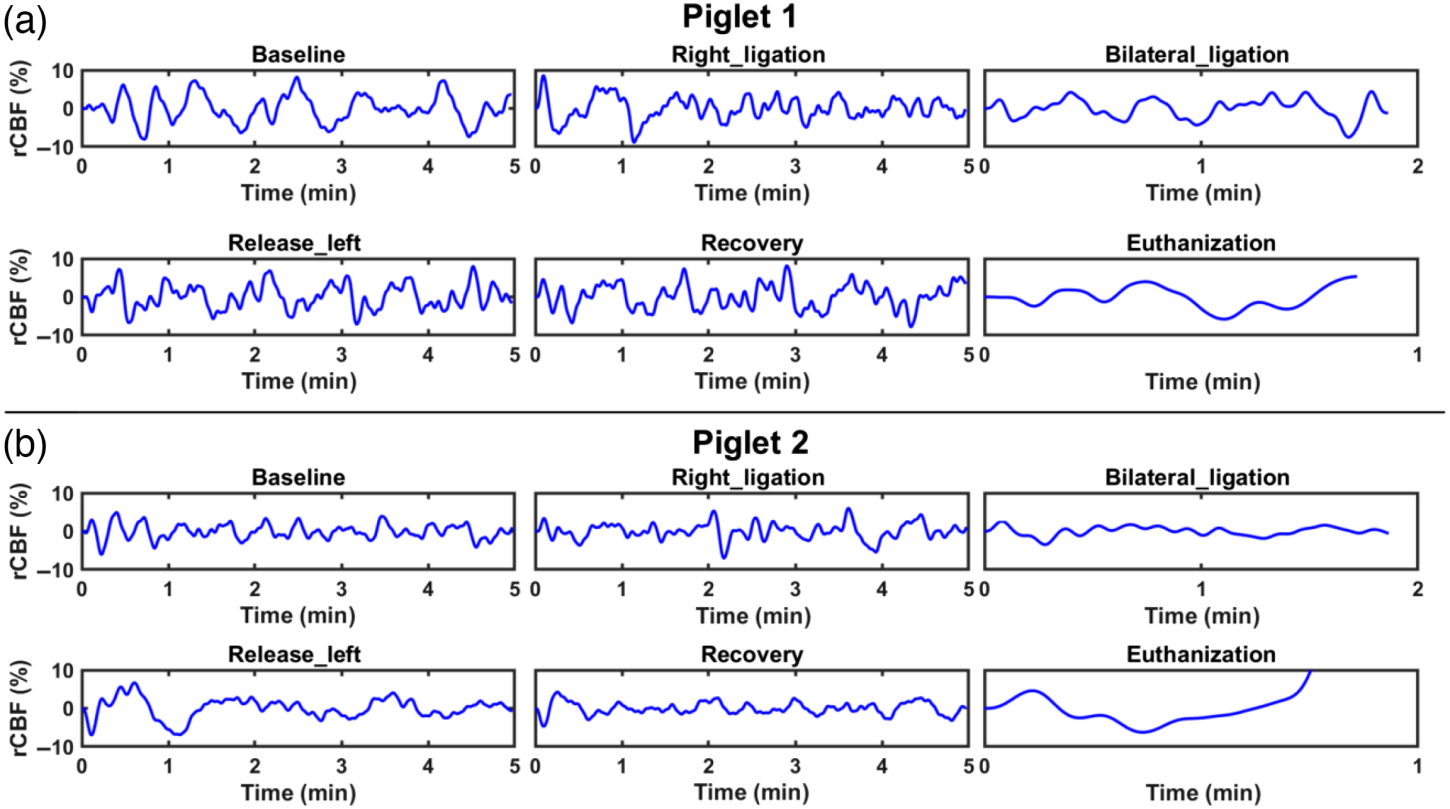
Detrended and bandpass filtered time course changes in rCBF over each interval for both piglets. (a) Piglet #1 and (b) piglet #2: time course changes in reanalyzed rCBF, from 5 mm beneath the skull, during the periods of: baseline, right CCA ligation, bilateral ligation (transient global ischemia), releasing of left ligation, releasing of both left and right ligations (recovery), and euthanasia (100% ${\mathrm{CO}}_{2}$). The detrended and bandpass filtered (0.01 to 0.1 Hz) rCBF represents spontaneous activity that occurred within the piglet’s brain.

Figure 5 shows the time course for rCBF during the baseline, right CCA occlusion, transient global ischemia, releasing the left CCA occlusion, releasing both left and right CCA occlusions, and euthanasia periods after detrending and LFO bandpass filtering (0.01 to 0.1 Hz). Hemodynamic fluctuations during transient global ischemia and euthanasia periods exhibited marked differences. This positive finding verified that LFOs as measured by the optimized scDCT have the potential for indicating abnormal cerebrovascular conditions.

### 3.3 PSD Changes in LFOs for Identification of Neurological Variations

**Fig. 6:**
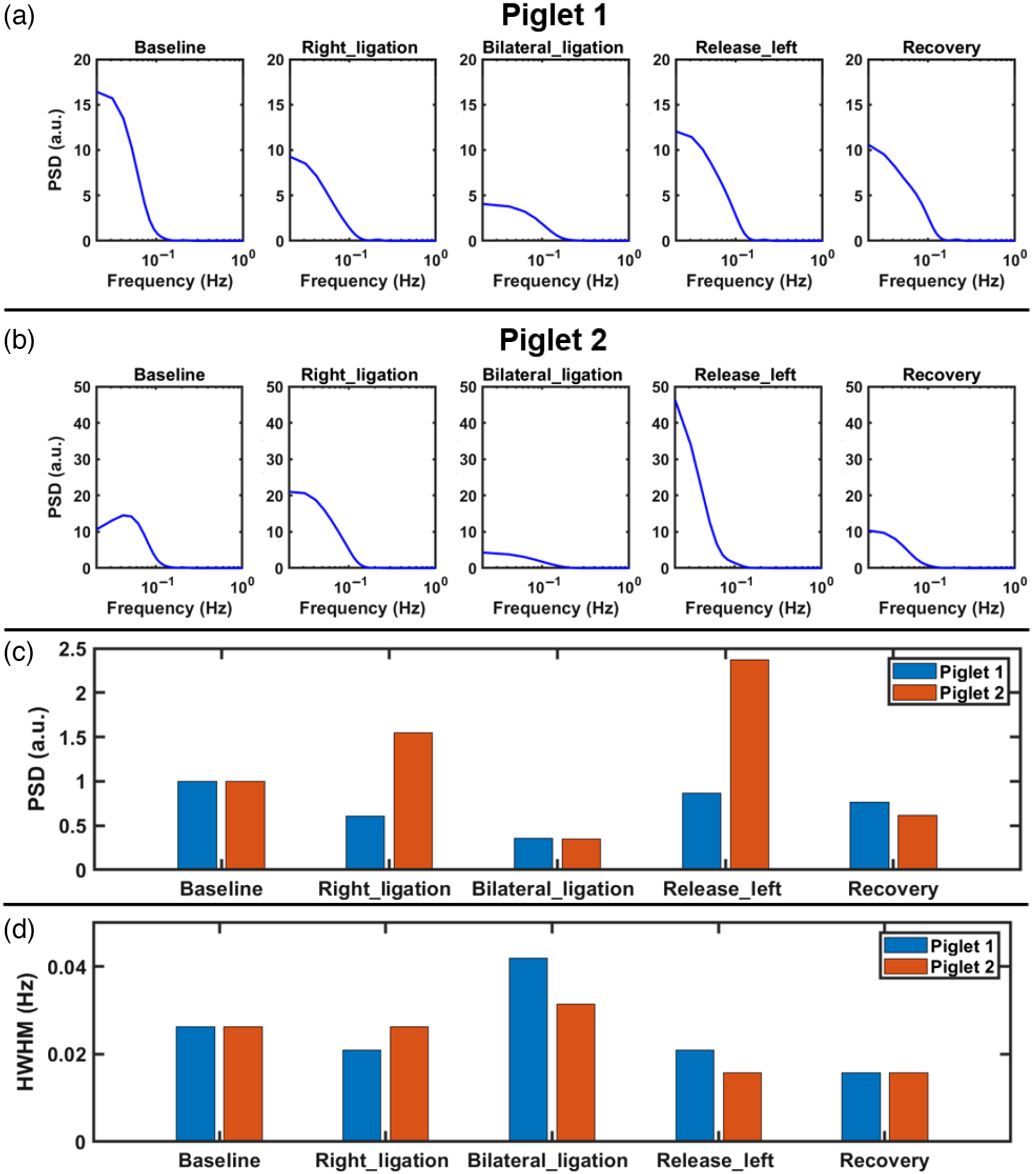
PSD analysis of LFOs in both piglets. (a), (b) Normalized PSD distributions from piglet #1 and piglet #2, respectively. PSDs were normalized to the AUC of the baseline PSD. (c), (d) PSD and HWHM changes during different phases of transient global cerebral ischemia from piglet #1 and piglet #2, respectively.

To better characterize the potential of LFOs as a measure of abnormal neurological status, we calculated changes in PSD within the LFO range. Figures 6(a) and 6(b) show normalized PSD distributions for both piglets. During the period of bilateral ligation, PSD decreased to ∼35% of the baseline for both piglet #1 and piglet #2 [Fig. 6(c)]. Correspondingly, HWHM values increased from 0.026 Hz at the baseline to 0.042 Hz (1.6 times increase) and 0.031 Hz (1.2 times increase) in piglet #1 and piglet #2, respectively [Fig. 6(d)]. Alterations in PSD and HWHM are likely associated with impairments of autonomic nervous activities during transient global cerebral ischemia. After release of bilateral ligation, both PSD and HWHM recovered toward their baseline levels.

## 4 Discussion and Conclusions

NIR diffuse optical instrumentation capable of noninvasively penetrating the deep brain offers a unique opportunity for CBF and LFO analyses.^16^^,^^37^^,^^66^ Among various NIR instruments, noncontact scDCT enables 3D imaging of CBF with flexible FOV and S–D arrangements for use across a wide range of subjects with varied head sizes within a low-cost package.^48^^,^^49^^,^^52^^,^^54^^,^^55^^,^^57^^,^^62^ However, this system was hindered in extracting LFOs due to an insufficient temporal sampling rate. This study utilized a moving window 3D reconstruction method with parallel computations to facilitate LFO studies. With this modified scDCT technique, a powerful tool is made available for neurologic studies and neonatal applications. The results from the unique design and testing efforts in neonatal piglets are discussed below.

A moving window 3D reconstruction method was employed to increase the temporal resolution of scDCT [Fig. 2(g)]. As evidenced by the zoom-in results in Fig. 3(a) (piglet #1) and Fig. 3(d) (piglet #2), the moving window method introduced a higher temporal resolution (5 Hz) compared with the conventional method (0.2 Hz). More frequency components were observed, suggesting increased sensitivity to underlying physiological variations, such as LFOs, as opposed to random noises and to the generally aperiodic pattern from the conventional method. It should be noted that the moving window method increased the temporal resolution 25 times compared with the conventional method at the expense of only utilizing data from one updated source location to generate a new image. Nevertheless, we were able to successfully measure and identify the higher frequency changes occurring in the blood flow responses during the ligation tests. This is expected as ligations have a far-reaching influence on the brain tissues and underlying physiological signals.

Moreover, a 3D reconstruction algorithm using parallelization functionality in MATLAB was developed for rapid image reconstruction while maintaining reconstruction quality. The completion time leveraged advance resources, such as graphics processing units (GPUs), at the HPC. Technological advancements and their availability improve the accessibility and potency of these resources in the lab and clinic. Improvements in computation speed vary depending on factors such as mesh density, hardware specifics, and competing processes.

We created a perinatal disease model of transient global cerebral ischemia in neonatal piglets to test the ability of the scDCT technique in detecting alterations in rCBF and LFOs. The transient global cerebral ischemia impacted entire brain hemodynamics. Despite the limited number of subjects, the observed large variations in rCBF during transient global cerebral ischemia (Fig. 3) align with pathophysiological expectations and agree with previous study results.^48^^,^^54^^,^^57^ The individual discrepancies in rCBF amplitudes during transient global cerebral ischemia are likely due to individual heterogenous responses in rCBF and differences in pathological stresses.

Performing spectral analysis on rCBF data at the baseline, common physiological frequency peaks (i.e., 2.5 Hz heartbeat and 0.5 Hz respiratory rate) and LFOs (<0.1  Hz) were identified (Fig. 4). The high temporal resolution of optimized scDCT (i.e., 5 Hz) is faster than typical cardiac and respiratory rates in neonatal piglets, allowing for the removal of their contributions to LFOs using simple lowpass filters. Thus LFOs in the range of 0.01 to 0.1 Hz were isolated from the high sampling rCBF curves for the resting baseline, right CCA occlusion, transient global cerebral ischemia, releasing the left CCA, and recovery phase. There were apparent reductions in rCBF fluctuations in correspondence with the ligations and euthanasia (Fig. 5). The bilateral ligation still retained an oscillatory nature, whereas euthanasia took on a monotonic trend. Correspondingly, attenuations in LFOs during ligations and euthanasia were also observed (Fig. 6). These observations are consistent with other studies reporting alterations in temporal and/or spectral characteristics of spontaneous LFOs following an ischemic stroke. For example, FC OISI was applied to mice with transient middle cerebral artery occlusions.^15^ LFOs of cerebral hemodynamic signals in the region of the brain affected by an ischemic stroke exhibit reduced fluctuations and significant reductions in their power spectra.^15^ According to a study conducted using the HD-DOT,^16^ the power spectra of LFOs are markedly lower in an infant who suffered a left occipital stroke than healthy term-born infants.^16^

Although rCBF and LFOs responses to transient global cerebral ischemia were coupled in this study, power spectral analysis of the detrended and filtered rCBF (i.e., LFOs) provided new insights into the neurologic status of the brain.^67^ During transient global cerebral ischemia, PSD values reduced dramatically compared with their baselines [Fig. 6(e)], indicating possible impairments in autonomic nervous activities. Meanwhile, HWHM values increased [Fig. 6(d)], suggesting LFO shifts to higher frequencies. These frequency shifts may be related to cerebral autoregulation mechanisms in response to dramatic variations in rCBF during transient global cerebral ischemia. Previous studies have found that different frequency bands within the LFO range are associated with different regulatory mechanisms including metabolic, neurogenic, and myogenic regulations.^68^^–^^70^ Further investigations on more subjects are needed to draw solid conclusions based on these new findings in PSD and HWHM alterations.

We note that our innovative scDCT system can be further improved in the future to map the brain’s resting-state functional connectivity (rs-FC). The rs-FC can be assessed by quantifying temporal correlations in LFOs across different regions of the brain, which enables depicting the brain’s functional organization.^37^ As the moving window method only uses a local update of information, the ability to capture highly distributed changes over the brain requires greater customization and consideration. To address this, the sampling rate of the scDCT should be further increased to remove entirely artifactual signals unrelated to neural activity. They may manifest as structured noise that introduces spurious correlations in the data and thus influences results. Use of a high-quality CCD or sCMOS camera with faster sampling rate and higher sensitivity would improve data acquisition, detection sensitivity, and probing depth. It is possible to further reduce the sampling time by modifying the number of sources, distribution pattern of the sources, and simultaneously illuminating multiple sources at the same time in different brain regions. In the future, it is planned to combine rCBF measurement with rs-FC mapping as well as the power of LFOs to perform novel functional neuroimaging with scDCT devices.

Some limitations that were not otherwise mentioned exist. One such limitation is that the optical properties (e.g., absorption and scattering coefficients) are known to have an influence on rCBF calculations.^71^ Optical properties are often assumed from the literature. Recently, a two-step fitting algorithm successfully extracted tissue absorption and blood flow simultaneously using the scDCT arrangement.^72^ This solution can assist in improving flow reconstruction accuracy. The utilization of multiple wavelengths of source light makes it possible to measure CBF as well as cerebral oxygenation and derivatives (e.g., tissue oxidative metabolism).^71^^,^^73^^,^^74^ By providing a more comprehensive assay, which includes fluctuations in hemoglobin concentrations ($\left[{\mathrm{HbO}}_{2}\right]$ and [Hb]) in addition to rCBF, it may be possible to clarify the disease-related hemodynamic and metabolic mechanisms involved in the disruption of brain networks as a result of disease. Also we note that the PSD interpretation of LFOs used here is only one approach and other options exist and will be studied for appropriateness. For example, the normalization of LFOs to the full frequency range has been used in fMRI to increase sensitivity and specificity.^75^

In this paper, the design of a high-speed scDCT imaging and reconstruction technique successfully increased effective temporal sampling, reconstructed full 3D data sets quickly, filtered non-LFO and undesired signal contributions, extracted LFOs from deep brain CBF, and characterized the LFOs through PSD throughout CCA ligation phases on neonatal piglets. The results provide a basis for scDCT instrument inclusion and applicability in the growing area of LFO analysis while bringing with it many inherent advantages for neurologic studies and potential clinical applications. Further studies with a greater number of subjects and better localization of neurological events and responses (e.g., mapping rs-FC) are needed to ensure these findings and conclusions in this pilot study.
